# Basic Differences and Most Common Findings in Ultrasound Examinations of Musculoskeletal System in Children: A Narrative Literature Review

**DOI:** 10.3390/healthcare10102010

**Published:** 2022-10-12

**Authors:** Tomasz Poboży, Wojciech Konarski, Karolina Piotrowska-Lis, Julia Domańska, Kamil Poboży, Maciej Kielar

**Affiliations:** 1 Department of Orthopedic Surgery, Ciechanów Hospital, 06-400 Ciechanów, Poland; 2 Central Clinical Hospital of the Ministry of Interior and Administration in Warsaw, 02-507 Warsaw, Poland; 3 Faculty of Medicine, Medical University of Warsaw, 01-938 Warsaw, Poland; 4 Surgery Clinic of Medical Department, Lazarski High School, 02-662 Warsaw, Poland

**Keywords:** ultrasound, pediatric sonoanatomy, coxitis, Baker’s cyst, ultrasonography, septic necrosis, JIA, arthritis, spondyloarthropathies

## Abstract

We present basic differences in the musculoskeletal ultrasound examinations between adults and children. Examiners who deal with adults on a daily basis have shared concerns about examining children. Such concerns may arise from the different approach to child ultrasounds, but they also come from differences in anatomical characteristics according to developmental age. We discuss the presence of growth plates, as well as non-mineralized parts of the bones. We also refer to the pathologies most often found in ultrasounds in early developmental stages. In the PubMed database, the set of keywords: “msk ultrasound in children”, “pediatric msk sonoanatomy”, “coxitis fugax”, “pediatric Baker’s cyst”, “Baker’s cyst ultrasonography”, “bone septic necrosis in ultrasonography”, “ultrasonography in juvenile idiopathic arthritis”, and “ultrasonography in juvenile spondyloarthropathies”, was used to identify a total of 1657 results, from which 54 was selected to be included in the article. We discuss the problem of osteochondritis dissecans, Osgood-Schlatter disease, examples of ligament injuries (especially in relation to the knee and ankle joints), exfoliation of growth cartilages, osteochondroma, exudates and inflammations affecting joints, and Baker’s cysts. In this way, we have collected useful information about the most common diseases of the musculoskeletal system in children.

## 1. Introduction

Ultrasound (US) examinations of the musculoskeletal (MSK) system is valuable in the diagnostics of diseases of the joints, ligaments, and muscles [1,2]. There are some differences in the anatomical structure of the musculoskeletal system in children and adults. Hence, examining children can be problematic for physicians who are routinely involved in the examination of adults. In this study, we highlight these differences and discuss the most common pathologies found in children. Our target is to summarize the basic differences in the approach to examining a child versus an adult. We also emphasize the differences in the equipment used during the examination.

Although there are many commonalities between conducting an ultrasound examination on a child and an adult, there are also differences. These differences exist for both healthy individuals and in individuals with pathologies.

It should be noted that there are pathologies that are typical to a specific developmental age. There are also some abnormalities that can have similar symptoms in children and adults, but the causes of these symptoms are completely different.

Chronic inflammatory diseases are different in children and adults, but their diagnosis is based on finding the same manifestations, such as synovitis, tenosynovitis, cartilage damage, bone changes, and enthesopathy.

## 2. Materials and Methods

We conducted a narrative literature review to summarize available knowledge about differences in musculoskeletal US examinations of children and adults, and about the most common pathological findings in children. Studies describing both differences and most common pathological findings in musculoskeletal ultrasonography in children were included. We excluded papers discussing rare pathologies or pathologies unrelated to the musculoskeletal system. We prepared a narrative synthesis of relevant papers identified in the PubMed database. The following set of keywords was used: “msk ultrasound in children”, “pediatric msk sonoanathomy”, “coxitis fugax”, “pediatric Baker’s cyst”, “Baker’s cyst ultrasonography”, “bone septic necrosis in ultrasonography”, “ultrasonography in juvenile idiopathic arthritis”, and “ultrasonography in juvenile spondyloarthropathies”. In the case of the last two phrases, we only collected results from the last 6 years due to the large number of the results. We identified a total of 1657 results. Overall, 54 papers on this subject were included. Papers suitable for inclusion in the review also had their references screened to identify relevant papers.

## 3. Results

### 3.1. The Differences in Musculoskeletal US Examination in Children and Adults

#### 3.1.1. The Differences between the Approach to Children and Adults: The Basic Requirements for the Musculoskeletal Ultrasound in Children

Ultrasound examination of the musculoskeletal system in children is slightly different than in adults, but it is not a more difficult examination. We focus on these differences in US examinations.

First, it is important to pay attention to the technical aspects and communication with the patient. During child examinations, the presence of a legal guardian is essential.

One of the advantages of an ultrasound examination (compared with MRI or X-ray) is the direct contact between the doctor and patient. During the examination, it is possible to take a history and perform a physical examination of the reported symptoms. In the case of young children, their medical history is taken from their parents. Sometimes, the examination may be challenging due to the restlessness of the child and their fear of the examination. It is necessary to try to minimize stressful factors. For some children, the sight of a person in a white apron may be such a factor; hence, we propose considering whether an apron is necessary. Whenever possible, the child should maintain physical contact with their parent during the examination. Many clinicians prefer to perform US examination in a darkened environment, although it is questioned if this truly improves the ability to distinguish different gray levels on the screen [3]. We suggest dimming the light only after the child has become accustomed to the new environment. The child should be informed about how the examination will progress, even if it seems that the child is too young to understand. It is also necessary to talk to child as much as possible during the examination [4]. 

Depending on the type of examination, we recommend considering performing the examination while the child is sitting on their caregiver’s lap. The doctor can introduce the probe to the child, e.g., by putting it in child’s hand before the examination.

The ultrasonography is painless, but using cold gel may be unpleasant and cause protest from a young patient. Remember to use gel warmers (many modern devices are equipped with heaters). The examination should be as efficient as possible and examination time should be reduced to a minimum. 

Children are not always able to indicate the localization of pain. It is commonly observed with hip joint diseases. In transient synovitis or slipped capital femoral epiphysis, children often complain of pain in the knee area. 

When a child is referred for an ultrasound examination of the knee joint, we suggest examining not only the knee joint, but also the hip joint, especially if the pathology has not been found in the knee joint.

Due to the relatively small size of the child’s body, the tissues of the musculoskeletal system are localized more superficially. Thus, linear probes of high frequency are recommended when performing the US examination.

Physicians used short probes (“hockey stick” or regular linear probes) in children more often than in adults. The most often used probes in our review had the following specifications: linear, 3–19 MHz, 192 element, using single-crystal technology with a 38 mm bandwidth. Some figures in this review were obtained with previously used 7–18 MHz probes. For examination of small and very superficial tissues, or even relatively larger joints in newborns, we recommend the compact linear probe, 10–25 MHz, bandwidth 25 mm or a standoff gel pad or a warm water bath—especially in regions with a curved contour. In older children, a probe of a lower frequency (e.g., 3–12 MHz) is often sufficient for examining deeper regions. When assessing increased flow, we prefer the Power Doppler option and Micro Flow imaging option, bearing in mind that it is better to use the normal Doppler option set to a low speed in some situations (e.g., when the child is excessively mobile, performing Power Doppler tests with high sensitivity may result in a large amount of artifacts). When using Doppler options in children, care should be taken not to exert too much pressure so as not to affect the results of the examination by closing the lumen of the vessels, which are more compressible than in adults.

In our practice, we try to use the latest technologies. The ultrasound scanner we currently use, the Alpinion X-CUBE 90, is equipped with the following technologies:

- SRI (Speckle Reduction Filter)—software reducing “speckle” noise in the image;
- SCI (Spatial Compounding Imaging), the so-called “cross imaging”;
- Edge Enhance—edge filters allowing to sharpen the boundaries between the tissues;
- THI (Tissue Harmonic Imaging)—harmonic imaging, which uses the second harmonic of the received signal to create an image, increasing the contrast resolution of received images;
- Inversion—harmonic imaging with inverted pulse. In addition to the main ultrasonic wave pulse, an impulse in the opposite phase is generated, which allows “natural” suppression of the reflected signal components related to the noise in the image. A much higher contrast resolution of the received images can be achieved;
- Single-Crystal—technology of probe construction that increases the frequency response, which in practice means it has a much higher sensitivity to a wider frequency range, and allows the probe to be used for both superficial and deep structures.

#### 3.1.2. The Anatomical Causes of Differences in US Examination of the MSK System in Children and Adults

The differences between children and adults in the US examination of the musculoskeletal system mainly have to do with joints, periarticular areas, and ligaments. 

Children’s joints have the same components as those of adults. The main difference is the presence of growth plates in children and the fact that the epiphyses in children are partially or completely composed of hyaline cartilage [5,6]. The extent to which the bone is made of hyaline cartilage depends on the age of child and specific anatomical area. 

In neonates, the femoral head is completely cartilaginous. The ossification nucleus appears between 4 and 10 months of age (usually around 6 months of age), and the ossification nucleus of the greater femoral trochanter usually appears between 2–6 years of age [7,8]. 

Ossification nuclei appear at different ages. With regard to the distal femoral epiphysis, it generates between 3 and 6 months, and proximal femoral epiphysis at 2–7 months of age [9,10,11]. The ossification nucleus of the tibial tuberosity generates between 10 to 12 years of age. At the age of 2 to 4 years the ossification nucleus of the proximal fibula appears, whereas the patella usually consists of one to three nuclei that arise at the age of 3–5 years [9]. 

In the case of the elbow joint, the ossification nuclei appear in the following order: ossification nucleus of the humeral head at 1 year of age, radial head at 3 years, medial epicondyle at 5 years, trochlea of humerus at 7 years, olecranon at 9 years, and lateral epicondyle of the humerus at 11 years of age [12]. 

Regarding the humerus, the sequence of appearance at the proximal end of the humerus is important. The ossification nucleus of the humeral head arises at the age of 1–6 months of age, the nucleus of ossification of the greater tubercle at 1 year of age, and the lesser tubercle between ages 3–5 [13]. 

We believe that it is not important to remember exactly when and where the ossification nuclei appear, but to bear in mind that the development of articular surface is a dynamic process.

The significant part of the epiphysis consists of hyaline cartilage. During examination of the cartilage, it is not possible to assess where the is boundary between articular cartilage and the deeper tissue that will become a subchondral layer of the bone. 

**Figure 1:**
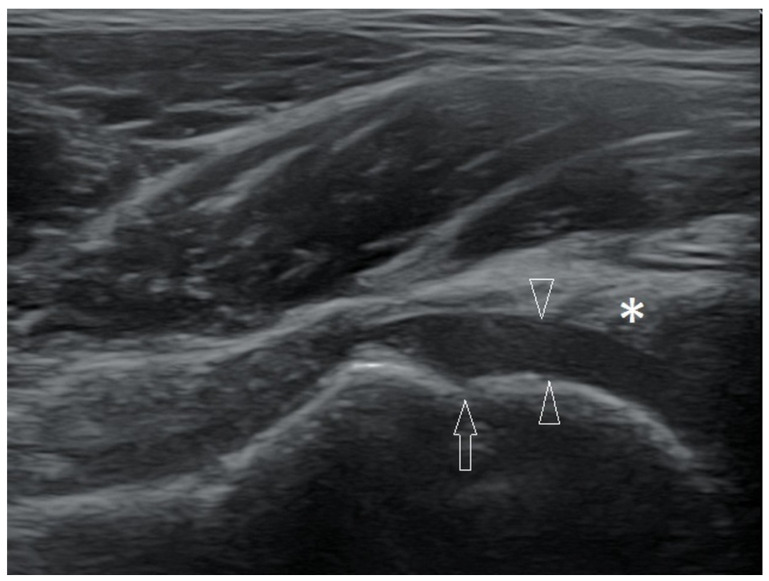
Longitudinal scan of the hip joint of a 5-year-old girl. Between the arrowheads—hyaline cartilage of the femoral head; arrow—level of the growth plate of the femoral head; asterisk—the labrum. Linear probe 3–12 MHz.

Considering the above, we recommend using the term hyaline cartilage and not articular cartilage in the description of a child’s joint examination. An example of a thick layer of hyaline cartilage on the femoral head of a 5-year-old girl is shown in Figure 1.

**Figure 2:**
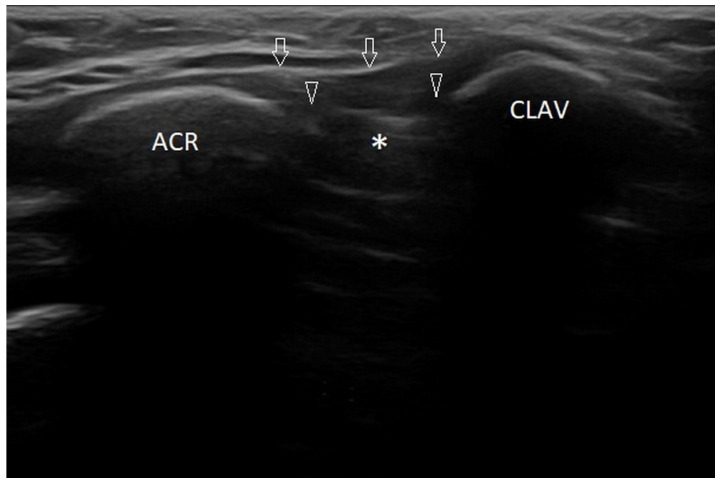
Frontal plane scan of the right acromioclavicular joint of 11-year-old boy. ACR—acromion; CLAV—clavicula; arrowheads—cartilaginous ends of acromion (left side of the image) and clavicula (right side of the image). Linear probe 3–12 MHz.

The articular ends of bones are made of anechoic or hypoechoic hyaline cartilage, so they may give the impression of widening the articular surface; thus, it is important not to confuse physiology with pathology. Figure 2 demonstrates an example of a healthy acromioclavicular joint in an 11-year-old boy.

**Figure 3:**
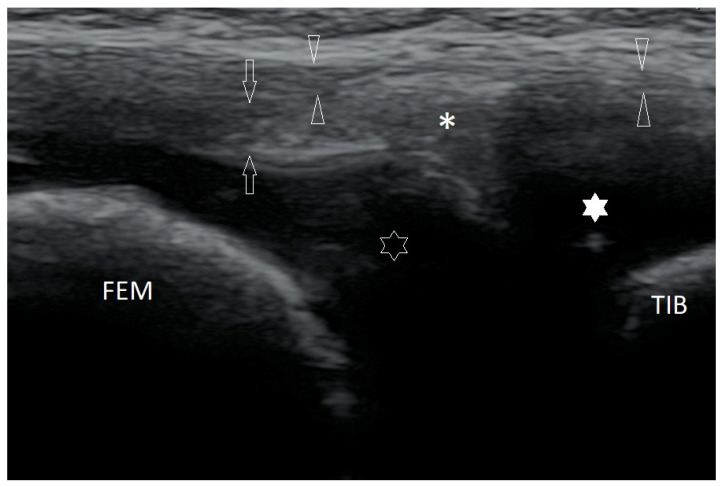
Longitudinal scan of the medial part of the right knee joint of a 5-year-old girl. FEM—ossified part of the medial femoral condyle (MFC); TIB—ossified part of the medial tibial condyle (MTC); hollow star—cartilaginous part of MFC; white star—cartilaginous part of MTC; asterisk—medial meniscus; arrows—deep part of medial collateral ligament (a meniscal-femoral ligament); arrowheads—the superficial (proper) part of the medial collateral ligament. Linear probe 3–12 MHz.

Assessment of articular structures is simpler or even more reliable when epiphyses are not completely ossified. For example, the echogenic structure of the meniscus in the knee joint contrasts perfectly with the anechoic hyaline cartilage, which means that it is possible to visualize the menisci at their full length, including the area around the horns of the menisci (Figure 3). The patella begins to ossify between ages 3–5 [9]; it allows better assessment of the cartilaginous epiphysis of the distal femur than in adults.

**Figure 4:**
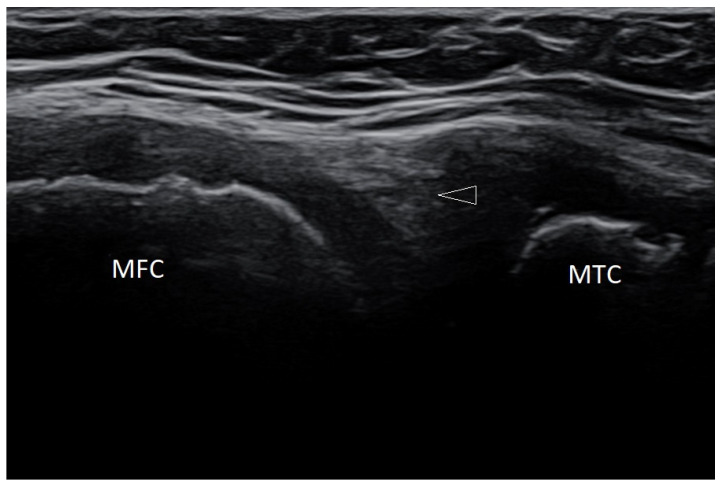
Longitudinal scan of the posterior-medial knee of a 10-year-old boy. MFC—medial femoral condyle; MTC—medial tibia; arrowhead—hypoechoic focus in the posterior part of the medial meniscus, which should be considered normal (physiologically).

When assessing the menisci in children, it should be remembered that the blood vessels and nerve endings penetrate the area of the posterior horns of the menisci; therefore, it is normal that the hypoechoic area can be observed there. (Figure 4).

It should be known that damage to the meniscus in children is less common than in adults [14].

Some conditions are normal in children but could be considered pathological in adults. In young children, a small amount of fluid in a joint is not a pathology. When assessing the fluid, it is necessary not to exert too much pressure on the probe, as the fluid-filled areas are more compressible in children than in adults, which may affect the outcome of the examination. It should be noted that physicians may have more difficulty distinguishing (or establishing a boundary) between fluid, thick epiphyseal cartilage, and synovium in children. The fluid may be of different echogenicity, undergoes redistribution under the infiltrate, and there are no vessels inside. The synovial membrane may also differ in echogenicity, is slightly susceptible to pressure, and may or may not show signs of increased vascularization.

The epiphyseal cartilage is anechoic or hypoechoic, is not compressible, and the individual vessels are often visible, especially in very young children.

During ultrasound examinations, it is important to remember about the presence of secondary ossification centers within the cartilaginous epiphyses in young children. Uneven margins of the ossification center is a common feature and should not be considered a pathology.

Focal cortical discontinuity at the site of an unfused physis should also not be mistaken for pathological conditions.

**Figure 5:**
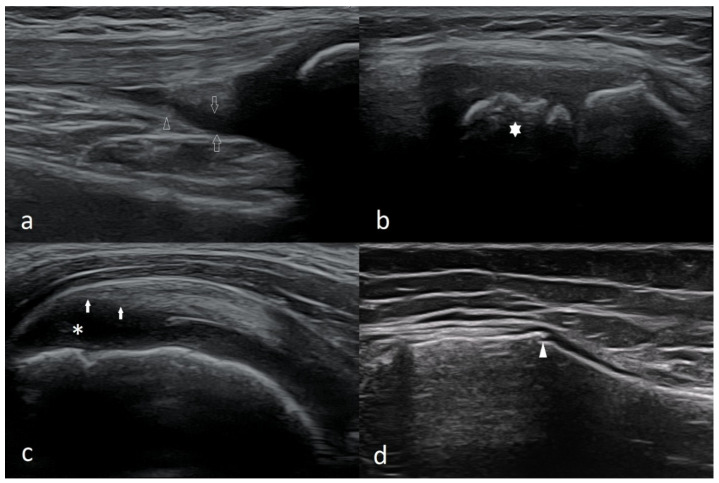
Examples of images of healthy children that could indicate a pathology in adults. (**a**) Suprapatellar recess in a healthy 5-year-old girl. Linear probe 3–12 MHz. Empty arrows—small amount of fluid; open arrowhead—small synovial fold. (**b**) Normal secondary ossification center of the medial tibial condyle (star) of a 9-year-old boy. The visible unevenness of the ossification center is not a pathology. Linear probe 3–12 MHz. (**c**) Attachment of the subscapularis tendon to the non-ossified lesser humeral tubercle (asterisk). Arrows—where tendon meets the outer surface of the tubercle. Linear probe 3–12 MHz. (**d**) Unevenness on the medial surface of the tibia in a 16-year-old girl in the place where there was a growth plate (between the proximal epiphysis and the shaft). Linear probe 3–19 MHz.

In older children, growth plate irregularities remain on the bone surface, which is not observed in adults. Such irregularities should not be mistaken for the joint space, fractures, or marginal erosion because of the typical location between the epiphysis and metaphysis (Figure 5) [15,16].

**Figure 6:**
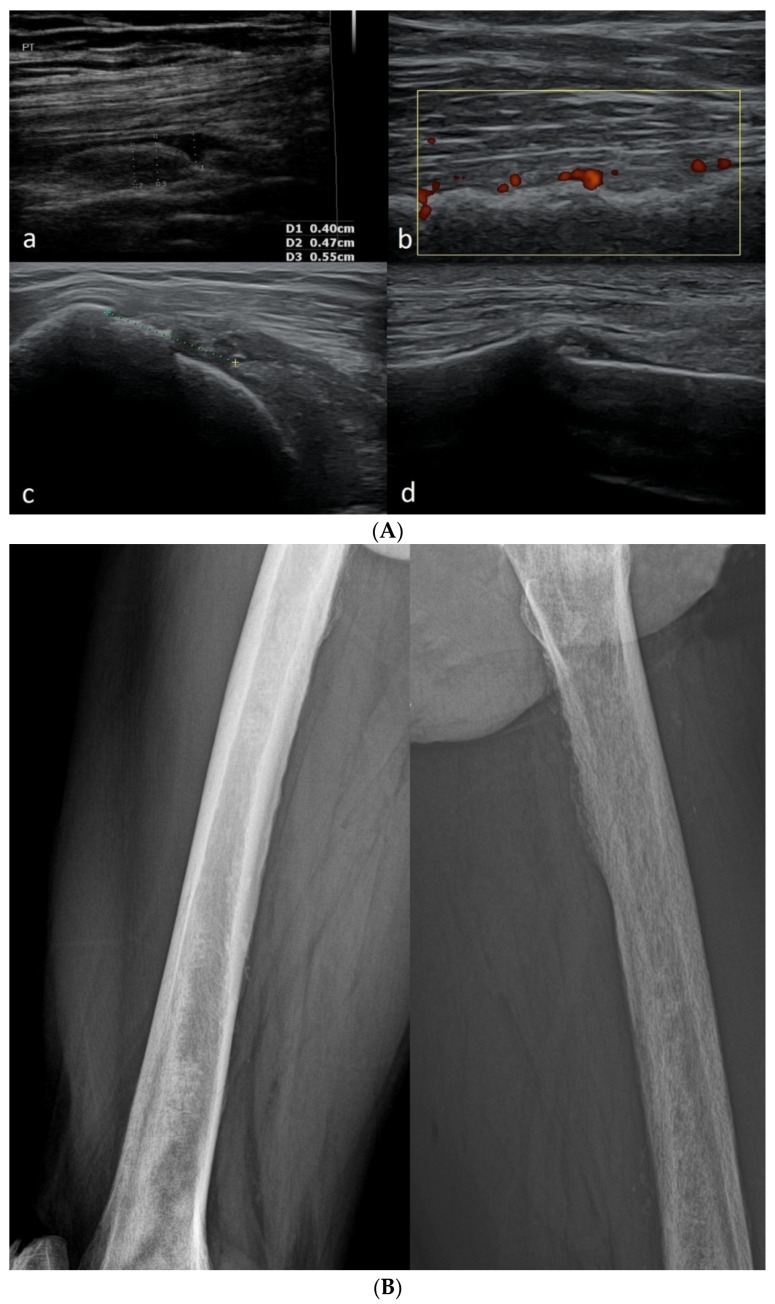

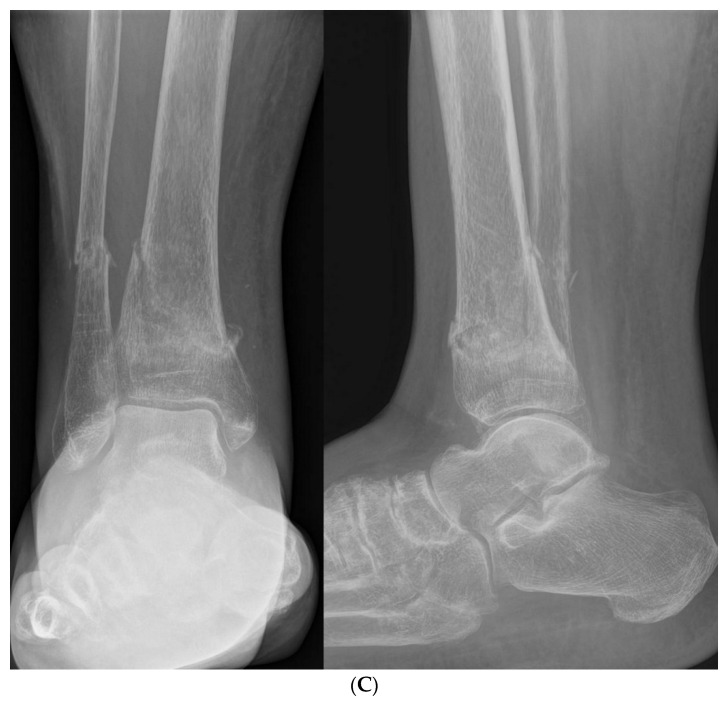
(**A**) Examples of pathological findings in adults (in comparison with the normal conditions in children from Figure 5). (**a**) Suprapatellar recess of a 48-year-old male with moderately severe inflammation due to gout. Visible folds of thickened synovium and a moderately increased amount of fluid. Linear probe 6–12 MHz. (**b**) Power Doppler image showing an uneven cortical layer of the femur in a 75-year-old male with multiple metastases of prostate cancer in the bones (the patient did not know about his disease until the day of ultrasound examination of the limbs). Visible increased vascularization of the periosteum. Linear probe 3–12 MHz. (**c**) Damage to the subscapularis tendon in a 58-year-old male; the distance to which the tendon has contracted is marked with a dotted line, the hypoechoic areas in the lesion zone correspond to traces of fluid and folds of the thickened synovial membrane inverting into the area of the lesion. 3–12 MHz linear probe. (**d**) Stress fracture of the tibia in a 61-year-old woman: visible discontinuity of the cortical layer, thickening of the periosteum, and callus formation. Linear probe 3–12 MHz. (**B**) X-ray images of the right (on the left) and left (on the right) femurs of a patient whose US images are presented in Figure 6(Ab). Visible osteolytic and osteosclerotic metastatic lesions in both femurs. (**C**) Anteroposterior and lateral X-ray images of the patient described in Figure 6(Ad). Patient presented with exacerbating pain in the distal 1/3 of the lower leg. There was no history of trauma.

Figure 6 presents pathological findings in adults that may correspond to normal findings in children.

**Figure 7:**
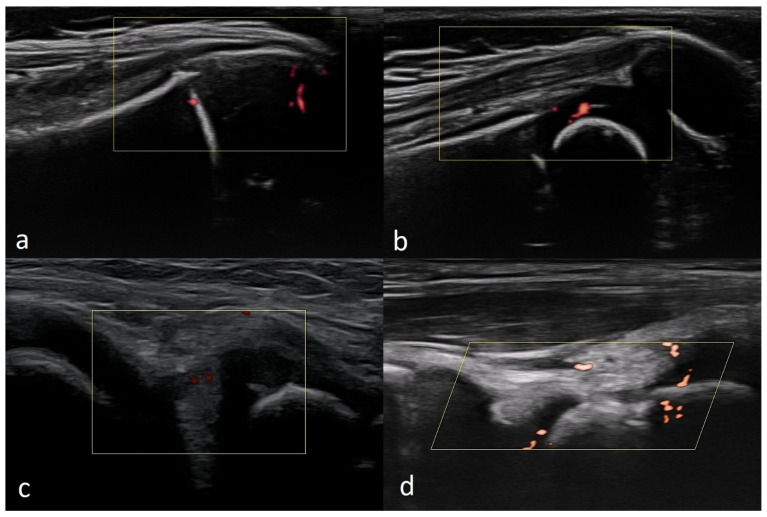
Examples of increased flow in Power Doppler imaging in children, which do not correspond to a pathology. (**a**) Frontal plane scan from the side—visible vessels within hyaline cartilage of the epiphysis of the femur of a 6-month-old boy. Linear probe 3–19 MHz. (**b**) Longitudinal scan of the suprapatellar recess of the same child—visible vessels near the growth plate. (**c**) Longitudinal scan of the posterior-medial part of the knee joint of a 9-year-old girl. Visible flow in vessels in the pericapsular zone of the posterior part of the medial meniscus. Linear probe 3–12 MHz. (**d**) Longitudinal scan of the anterior recess of the ankle joint of a 5-month-old boy. The visible signals are artifacts from excessive motion. Linear probe 10–25 MHz.

In US Doppler imaging, there may be an intra-articular/peri-articular Doppler signal in children that would be considered an abnormal finding in adults. A flow can be seen within the cartilaginous epiphyses, especially in young children up to the age of 3 (Figure 7a). This feature is also apparent in the growth plate area (Figure 7b).

In some areas, vessels are visible in a developmental age, then undergo atrophy and are replaced by the fibrous tissue (e.g., in the posterior horns of the menisci) (Figure 7c). 

Some anatomical variations may suggest some specific pathologies.

US examination gives the opportunity to immediately verify if the pathology also occurs on the opposite side (it is worth noting that some diseases may affect the joints symmetrically, such as the juvenile idiopathic arthritis).

In children, it is easier to obtain false-positive results showing an increased flow (as a consequence of excessive motion), particularly in a Power Doppler test (Figure 7d) (flash artifact) [15,17,18].

Basic differences in imaging of healthy tissues within the musculoskeletal system in children and adults are summarized in Table 1.

### 3.2. The Most Common Pathologies Encountered in the US Image in Children

#### 3.2.1. Osteochondritis Dissecans

**Figure 8:**
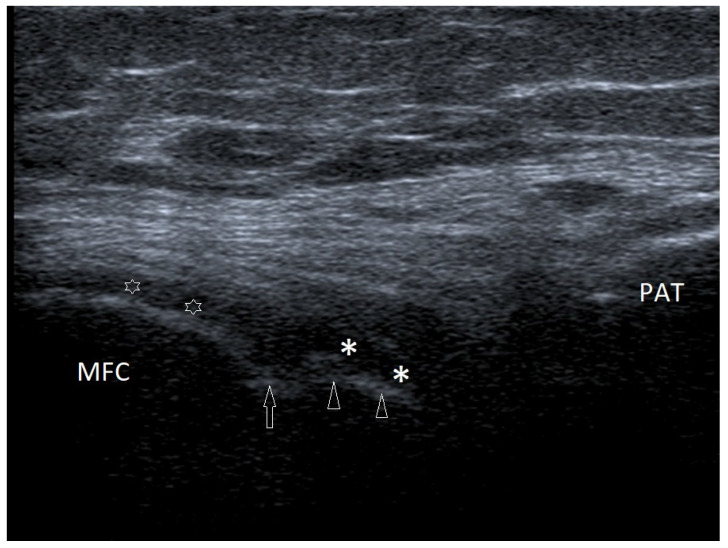
A case of OCD in a 15-year-old boy. Cross-section at the level of the medial femoral condyle (MFC); arrow—break in the subchondral bone at the edge of the OCD lesion; arrowheads—subchondral bone in the OCD zone; asterisks—swollen cartilage in the OCD zone; empty stars—cartilage covering the healthy part of the MFC, also with symptoms of slight swelling (increased echogenicity and slightly increased thickness); PAT—patella. Linear probe 7–18 MHz.

Osteochondritis dissecans (OCD) often affects boys over 12 years of age [19]. The lesions are typically located in the medial femoral condyle (MFC) in the femorotibial joint close to the intercondylar fossa [19,20]. This area is more difficult to access by ultrasound examination, as it is often covered by the patella (especially in children at age when the patella is ossified). OCD is diagnosed at various stages of advancement, sometimes in the initial stage, sometimes after the separation of the loose body. Figure 8 demonstrates a case of OCD in a 15-year-old boy in the early stage (grade II in the Clanton and DeLee classification), an interruption in the continuity of the subchondral bone and overlaying cartilage with preserved continuity, although the cartilage is swollen [21,22,23]. 

OCD may affect the other parts of the joint, including the surface of the lateral femoral condyle or the patella [24]. Changes in a location other than the MFC are mainly associated with past injuries.

OCD may also involve the ankle joint, usually located on the medial edge, the superior part of trochlea of talus, and is often related to the trauma [25,26,27]. 

The diagnosis is based on X-ray and/or MRI because this area is difficult to access in ultrasonography.

#### 3.2.2. Osgood–Schlatter Disease

Another common disease located in the knee joint is Osgood–Schlatter disease, which concerns the tibial tuberosity. Osgood–Schlatter is caused by repeated overload and chronic detachment of the apophysis of the tibial tubercle [28,29,30,31,32,33]. The disease usually occurs in children from ages 9 to 14, about four times more frequently in children active in sports [34,35,36]. In our practice, the disease mainly affects young athletes, mainly boys playing football, volleyball, or jogging. 

**Figure 9:**
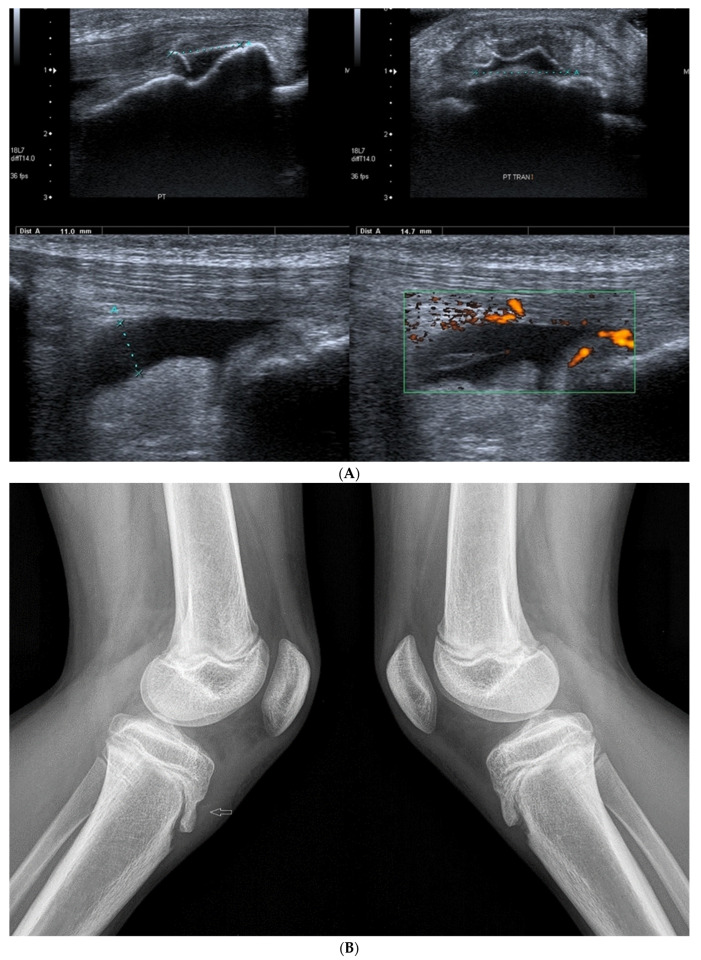
(**A**) The case of a 13-year-old girl in whom the symptoms of Osgood–Schlatter disease were clearly related to a sport injury (avulsion of the bony fragment from the tibial tuberosity). At the top, there are scans, longitudinal on the left and transverse on the right, with a marked thin layer of bone detached from the surface of the tibial tuberosity. At the bottom, two scans in the longitudinal section where there is an exudate in a deep subpatellar bursa. On the right, there is increased vascularization of the adjacent tissues (Power Doppler). Linear probe 7–18 MHz. (**B**) Lateral X-ray images of the patient from Figure 9A On the left—affected left knee joint, the arrow marks a small bony fragment detached from the tibial tuberosity. On the right—a healthy right knee joint.

Figure 9 presents the case of a 13-year-old girl who trains intensively in speed skating. 

The occurrence of symptoms has an evident traumatic background here; both the mechanism of the injury and the imaging tests indicate an avulsion of the fragment of the tibial tuberosity (the girl felt a sharp pain in the infrapatellar area during a sudden start of motion during training).

#### 3.2.3. Ligament Injuries

When assessing ligament or tendon attachments, their ends will usually connect with the anechoic area corresponding to non-calcified bone, not with the echogenic layer corresponding to the mature bone surface.

**Figure 10:**
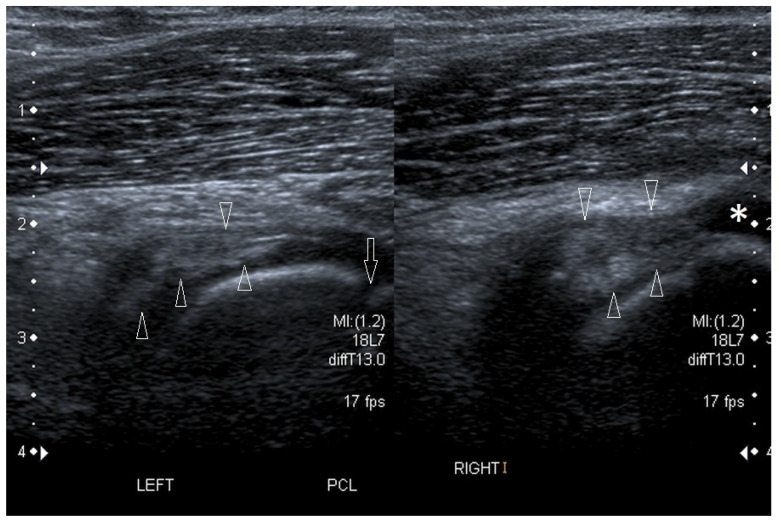
A case of posterior cruciate ligament (PCL) injury in a 15-year-old boy. Longitudinal scans through the middle part of the popliteal fossa. On the left—the image of healthy, undamaged PCL (arrowheads) in the left knee joint, the arrow indicates the level of growth plate. On the right—completely damaged, shrunken in the peripheral direction and doubled PCL (arrowheads); asterisk—fluid posterior to the proximal epiphysis of the tibia. Linear probe 7–18 MHz.

In children, injuries may occur in the same ligaments as in adults. For example, Figure 10 demonstrates a case of posterior cruciate ligament injury in a 15-year-old boy.

Some injuries occur more often than others. As in adults, the knee and ankle joints are damaged more often than other regions [37,38,39,40,41,42]. 

**Figure 11:**
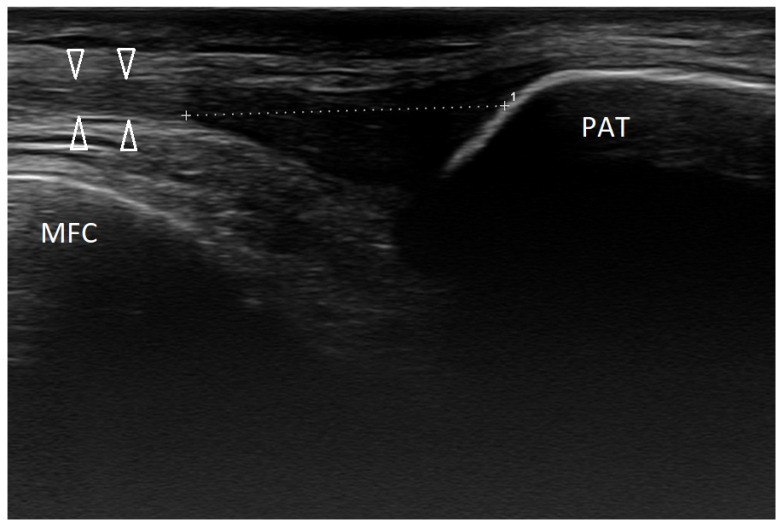
Hypoechoic scar formation (dotted line) in the medial patellofemoral ligament (MPFL) of the left knee joint. Probe placed along the course of the MPFL. Arrowheads—intact part of the MPFL. MFC—medial femoral condyle. PAT—patella. Linear probe 3–12 MHz.

In our practice, the most common injury in the knee joint involves damage to the medial patellofemoral ligament (Figure 11), sometimes with an avulsion of the bone fragment from the medial edge of the patella.

In the case of the ankle joint, as in adults, the injuries often concern the anterior talofibular ligament (ATFL), but often with avulsion of the bony fragment in children [43]. 

**Figure 12:**
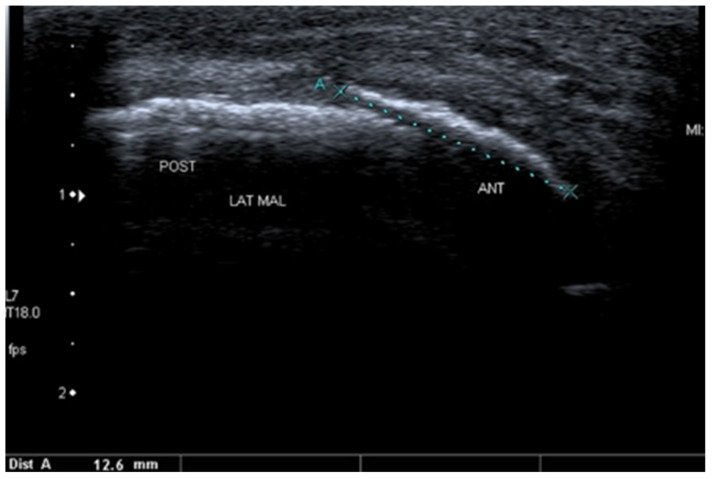
Avulsion of the proximal attachment of the anterior talofibular ligament (ATFL). The dotted line A marks the avulsed and anteriorly displaced fragment of the lateral malleolus (LM). POST—posterior; ANT—anterior part of the ankle. Probe positioned along course of the ATFL. Linear probe 7–18 MHz.

Figure 12 presents an avulsion of ATFL attachment to the lateral malleolus in a 10-year-old girl.

Compared with other joints (elbow joint, humeral joint, and carpal joint), fractures of bony ends (epiphyses and metaphyses) seem to be more frequent than ligament injuries. In the case of a relatively frequent trauma, such as subluxation of the radial head, patients are usually not referred for an US examination because either spontaneous reposition occurs, or the problem is clinically diagnosed (and/or radiologically by X-ray), and easily treated without permanent consequences.

#### 3.2.4. Exfoliation of Growth Plates

**Figure 13:**
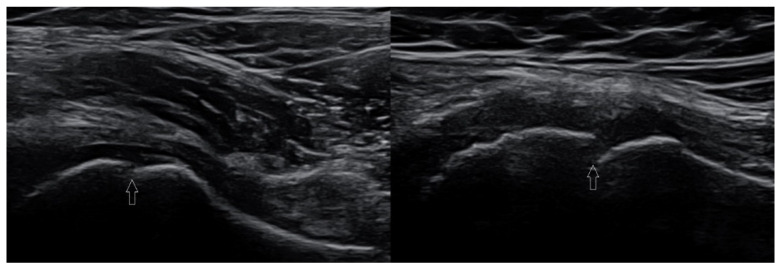
Image of the normal growth plates of the proximal end of the femur. Left arrow—growth plate of the femoral head (probe positioned along the long axis of the femoral neck); right arrow—growth plate of the greater trochanter (long axis view in the frontal plane). Linear probe 3–12 MHz.

Growth plates are visible on the border of the epiphysis and metaphysis during developmental ages. For example, Figure 13 shows the image of normal growth plates of the femoral head and greater trochanter in a 10-year-old boy. Their presence predisposes children to specific injuries such as exfoliation of the epiphysis.

Exfoliation of the growth plate often has a traumatic background. The diagnosis is usually based on physical examination and X-ray. One of the more serious clinical problems is a slipped capital femoral epiphysis. The diagnosis is also based on X-ray, but some signs can be visualized with an US examination. It is usually initiated by minor trauma and associated with obesity or endocrine disorders, such as hypothyroidism, hypopituitarism, and hypogonadism. It can also be the result of treatment with ionizing rays or chemotherapy. Slipped capital femoral epiphysis occurs more frequently in boys than in girls [44,45].

**Figure 14:**
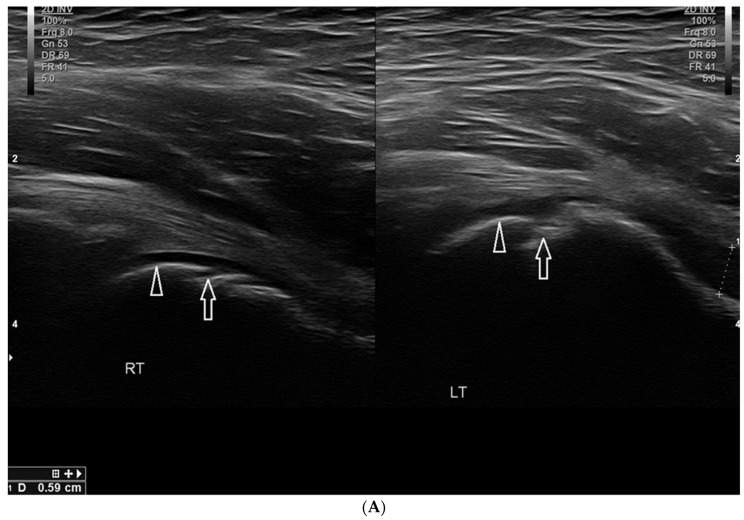

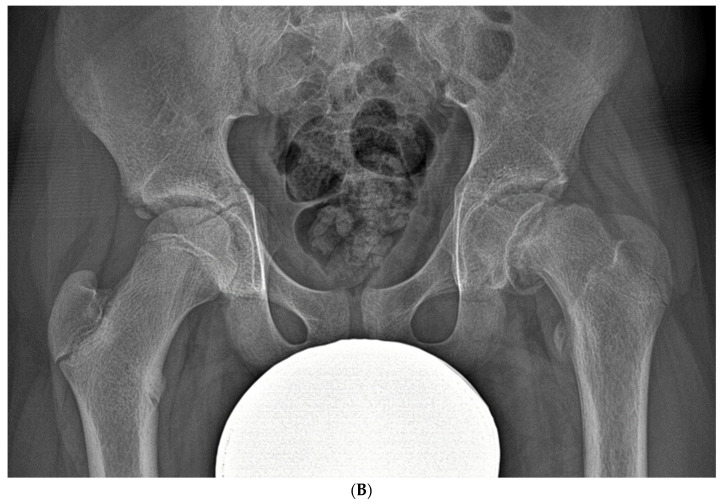
(**A**) A case of exfoliation of the femoral head in a 13-year-old boy. Images are in the plane of the long axis of the femoral neck. Image of healthy right hip joint (right); exfoliation of the left femoral head (left). Arrows—level of growth plate of the femoral head; arrowheads—proximal femoral epiphysis. Accompanying exudate (dotted line) is visible in the left hip joint. Linear probe 3–19 MHz. Figure 14. (**B**) X-ray image of the same patient. Visible exfoliation of the left femoral head.

Figure 14 presents slipped capital femoral epiphysis of the left femur in a 13-year-old boy.

#### 3.2.5. Osteochondroma

The growth plate is a zone of an intense cell division, which is predisposed to the occurrence of malignant neoplasms in the epiphyses of children. 

The most frequently occurring types include: osteosarcoma (between 12 and 18 years of age) and Ewing’s sarcoma (about 15 years of age) [46,47]. 

**Figure 15:**
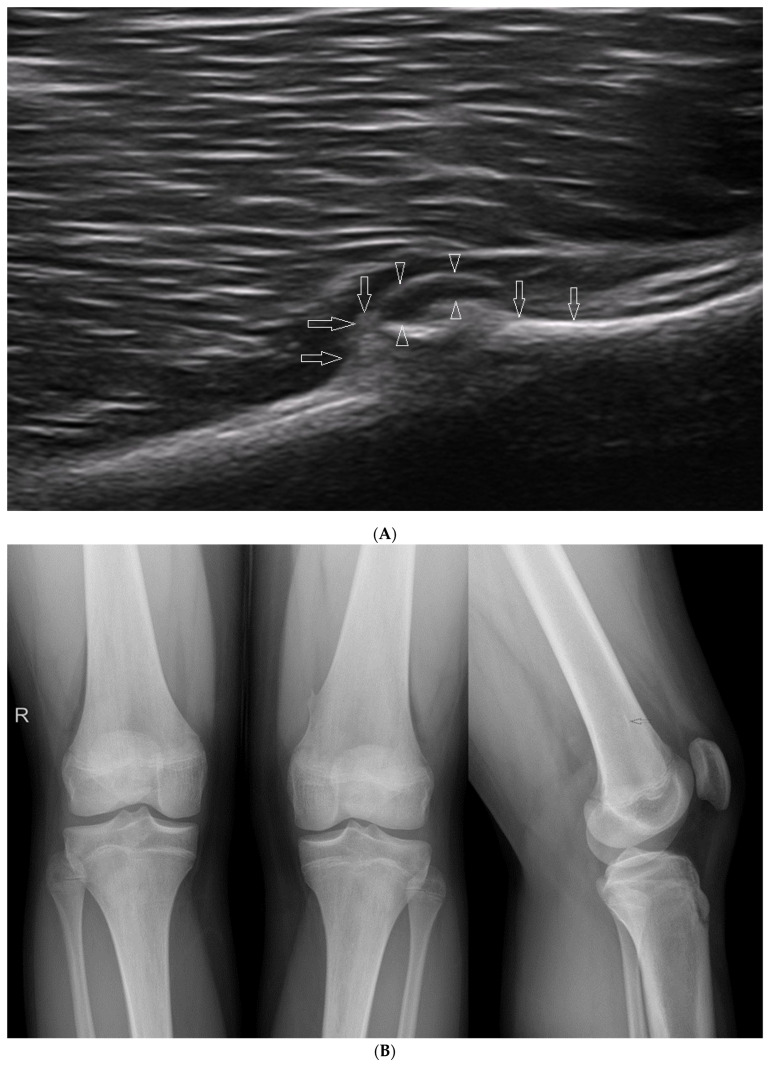
(**A**) An example of an osteochondroma exostosis of the left femur of a 16-year-old boy. Long axis view. Arrows—bony part of the exostosis; arrowheads—partly built of hyaline cartilage (anechoic). Linear probe 3–19 MHz. (**B**) Comparative anteroposterior X-ray image of the knee joints from the same patient and lateral image of the left knee joint, with visible osteochondroma of the left femur. Small empty arrow—longitudinal shadow corresponding to the exostosis on the lateral radiograph.

In clinical practice, however, the physicians usually deal with mild changes, such as osteochondromas that are typically also located near the growth plate [48]. An example is presented in Figure 15.

#### 3.2.6. Exudates and Inflammations Affecting Joints

Although the exudate in joints are the easiest to diagnose, we present them at the end of the article. The increased fluid may be observed after traumas or in inflammatory diseases (e.g., juvenile idiopathic arthritis). A thickened synovial membrane with intensively vascularization are characteristic of inflammatory diseases. 

**Figure 16:**
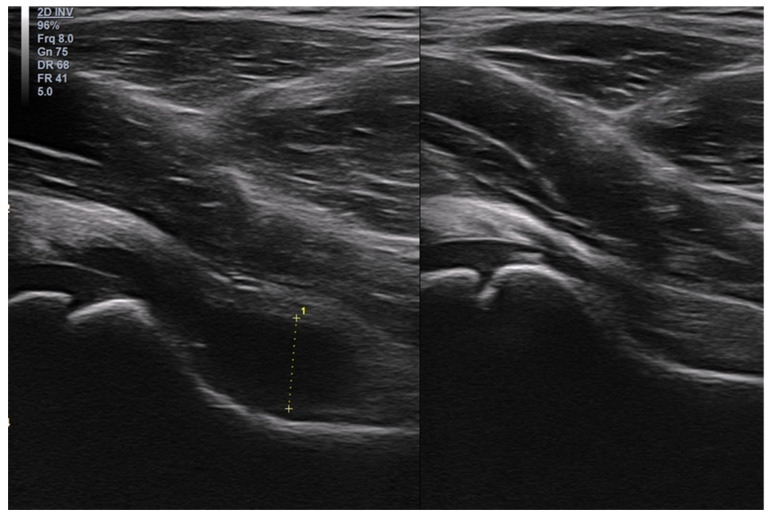
A case of transient inflammation of the right hip joint in a 5-year-old boy. Images in the plane of the long axis of the femoral neck. The exudate in the right hip joint is marked with a dotted line (**left** side of the image). A normal image of the healthy left hip joint (**right**). Linear probe 3–19 MHz.

Transient synovitis is an example of a mild disease with concomitant exudate. It is typical of developmental ages, mainly observed in children between 3 and 8 years old, and more often in boys. The pathology affects the hip joint and is commonly referred to as coxitis fugax. The etiology is unknown, but it may happen after a recent viral infection or trauma. The ultrasound examination shows an effusion in the articular cavity (Figure 16). Transient synovitis is a self-limited disease, and usually lasts 5 to 12 days [49].

However, it should be noted that the ultrasound examination is an additional test and is not considered to be a confirmed diagnosis. The complete clinical picture is important.

Articular exudate in a hip joint may occur in the course of other serious pathologies, such as Perthes disease, rheumatological diseases, or septic arthritis.

The role of ultrasonography in the diagnosis of juvenile idiopathic arthritis and other inflammatory diseases cannot be underestimated. Although contrast-enhanced magnetic resonance imaging is the standard reference imaging modality in the diagnosis of JIA, and X-ray allows a quick assessment of whether the physician is dealing with post-traumatic or nodular changes, the ultrasound enables a quick diagnosis without the use of radiation. It is usually easily accessible and highly mobile. US examination is often performed as the first imaging study at the early stages of diagnosis, but also plays an important role in monitoring treatment and determining remission. The role of ultrasound examinations in treatment is also important (interventional ultrasound), such as using US for joint aspiration, injections, etc. [15,50]. 

**Figure 17:**
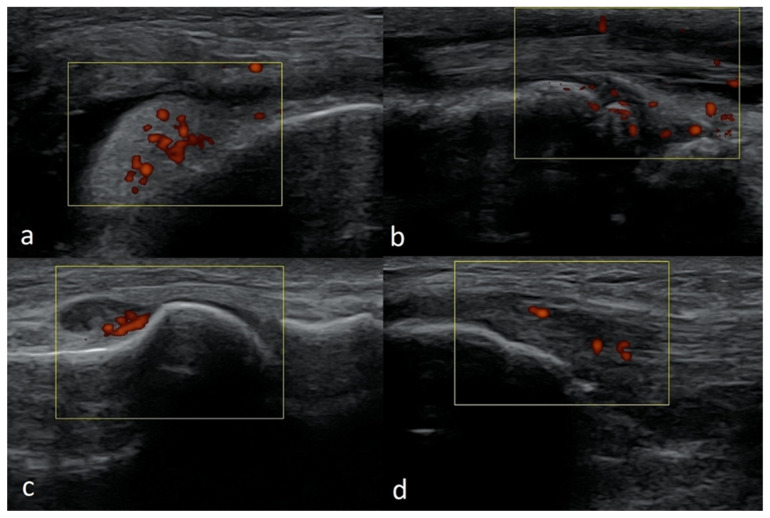
(**a**) Transverse scan through the lateral part of the suprapatellar recess in a 9-year-old girl with synovitis of the knee joint, showing visible synovial hypertrophy with increased vascularization. (**b**) Tenosynovitis of tibialis posterior at the level of the medial malleolus, with visible synovial thickening, hypervascularity, and increased amount of fluid around the tendon. (**c**) Synovitis of the I metatarsophalangeal joint in a 47-year-old female, showing increased amounts of fluid and a thickened, hypervascularised synovium. (**d**) Enthesitis of patellar tendon in a 37-year-old male, showing thickening, decreased echogenicity, and features of increased vascularization. Linear probe 3–12 MHz.

Returning to their role in diagnostics, US examinations are not specific. However, it allows one to identify the presence of inflammatory and post-inflammatory lesions, including synovitis, tenosynovitis, cartilage damage, bone changes, and enthesopathy—examples of these pathologies are presented in the Figure 17.

These are, of course, changes that can also be found in adults with rheumatoid arthritis at various stages of advancement. In children, it is especially important to recognize the features of inflammatory diseases at early stages so that they can start appropriate treatment before permanent tissue damage occur [15,51,52,53]. 

#### 3.2.7. Baker’s Cysts

In children, fluid may be accumulated in the joints but there may also be fluid reservoirs around the joints. These reservoirs are ganglions or Baker’s cyst. 

Similar to adults, ganglions are often observed in the wrist region. A cyst in the popliteal fossa in the location of the gastrocnemius bursa is a typical Baker’s cyst. 

In adults, exudates of this type appear secondary to other pathologies. These pathologies, such as chondromalacia or meniscus damage, lead to the appearance of exudate in the joint, and the fluid from the joint cavity flows though the continuity into the bursa, resulting in enlargement of the bursa [54]. 

**Figure 18:**
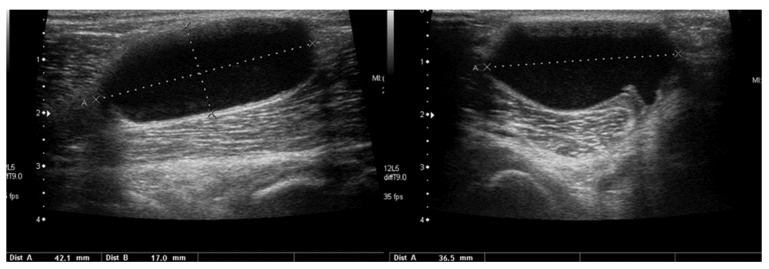
Longitudinal (**left**) and transverse (**right**) views of a Baker’s cyst in the right popliteal fossa of an 11-year-old boy.

In children, however, the Baker’s cyst is usually an isolated (primary) pathology (Figure 18). 

The differences in the US examination of musculoskeletal system in children and adults are discussed above. Examples of the most common pathologies are also introduced. In everyday practice, various pathologies within the musculoskeletal system must be taken into account. Table 2 summarizes the most dense pathologies in individual tissues and organs of the musculoskeletal system.

## 4. Discussion

In our experience, many doctors who perform orthopedic ultrasound examinations in adults tend to avoid performing such examinations in children. In our everyday practice, we often perform tests on children whose parents admit that they have been refused such tests at other clinics, where tests are performed mostly on adults.

Although it is obvious that US testing differs between a child and an adult, these differences are easy to apply and adapt to. First, an appropriate approach to children examinations is necessary. Also important are the differences in anatomy corresponding to the different developmental ages.

Another important thing to remember is how the musculoskeletal system develops. The epiphyses of an infant are separated from the shafts by growth plates and are made of hyaline cartilage. Within the cartilaginous epiphysis, secondary centers of ossification appear over time. Before the epiphyses are ossified, the presence of vessels within them can be detected. Vessels are also present in growth plates; such phenomena are not observed in adults. The presence of growth plates can lead to specific injuries such as exfoliation.

Many pathologies and post-traumatic lesions in children and adults look the same or similar. However, there are some medical conditions that are typical in children. In our study, we have discussed the most common ones. This is not an exhaustive list, and physicians may encounter less common conditions that are difficult to assess, when examining children. An example of such conditions may be neoplasms. It is important to remember that the task is not to make a histopathological diagnosis, because it is not possible to do so with US examinations. The focus should be to describe the lesion by determining its location, size, and echogenicity. The US can be used to determine echostructure and homogeneity and assess separation from the environment, looking for the possible presence of a capsule or measuring features of tissue infiltration, determining vascularization using Power Doppler or Micro-Flow tests. The US examination often constitutes a basis for further diagnostics. Hence, when identifying one pathology or another, physicians are often obliged to refer patients to the next test that should be performed.

The apprehension of misdiagnosing a serious pathology, such as a malignant tumor, may be one of the reasons why some researchers are reluctant to perform US in children. This is similar to diseases in the field of pediatric rheumatology. However, it is important to remember that, even in rheumatoid diseases, physicians do not have to (and sometimes should not) make the final diagnosis. The task of the examiner is to describe features of the inflammation, such as effusions, synovial thickening, synovial hypervascularity, or increased echogenicity of the synovium, indicating chronic inflammation or fibrosis. The diagnosis is made based on the entire clinical state, including medical history, physical examination, and laboratory tests. When it comes to imaging studies, the US cannot replace other tests. Contrast material through enhanced magnetic resonance imaging undoubtedly plays an irreplaceable role in the diagnosis of JIA and other rheumatoid diseases in children. The role of classical radiography is essential in the diagnosis of the MSK system.

X-ray plays a key role in recognizing post-traumatic changes, including fractures, dislocations, and exfoliation. Many other diseases also have their own characteristic image, whether they are benign conditions, such as cartilage and bone growth, cortical fibrous defect, enchondroma, and bone cysts, or malignant neoplastic lesions and many others.

The role of US as a fast, easily available, real-time, functional test that allows for comparative testing is well-established and has its proper place among other diagnostic methods.

## Figures and Tables

**Table 1:** Basic differences in imaging of healthy tissues within the musculoskeletal system in children and adults.

Assesed Structure	In Children	In Adults
Joint space	A possibility of the physiological presence of a small amount of fluid or small synovial folds	A possibility of physiologically moderately increased amount of fluid after physical activity or in the evening
Articular cartilage	Relatively thick layer of hyaline cartilage (the non-ossified part of the epiphysis), the possibility of existence of blood vessels in the cartilaginous epiphysis at a young age	A thin layer of articular cartilage, lack of vascularity
Growth cartilage	Present. The possibility of the presence of blood vessels in the area of growth cartilage. In adolescents—unevenness at the site of growth plates.	Not present. Unevenness on the bone surface is usually an indication of pathology.
Muscle and tendon attachments	Attach to non-ossified hypoechoic bone parts	Attach to calcified bones

**Table 2:** US assessment of the tissues within the musculoskeletal system and the most common pathologies found within these tissues in children.

Assessed Structures	Potential Pathologies
Subcutaneous tissue	Edema, postraumatic “hematomas”, discontinuity (Morel–Lavallée lesion)—rare in children, and tumors (lipomas, fibromas, and neurofibromas, such asVon Recklinghausen’s disease)
Fascia	Discontinuity, muscle hernias—usually coexisting with post-operative or traumatic scars, and desmoid fibromatosis
Muscles	Post-traumatic changes, breaks in continuity, “hematomas”, and tumors (a lesion with the features of a lipoma is benign if it occurs in the subcutaneous tissue; if it is visible within the muscles, at the depth of the fascia, it is likely to be a malignant (e.g., liposarcoma), even if it has well-defined edges and does not show features of hypervascularity)
Tendons	Tendon injuries, enthesopathies, enthesitis, tendinopathies, tenosynovitis, tendinitis secondary to tenosynovitis, and stenosing tenosynovitis (most frequent in children is the congenital trigger thumb)
Bone surface	Post-traumatic changes, features of fractures, local thickening of the periosteum, increased vascularization of the periosteum, and the presence of periosteal reactions, which may indicate the presence of osteomyelitis or neoplasms
Nerves	Pathologies of the peripheral nervous system in children are rare, compression syndromes are practically absent, and tumors, which include rare neuromas and fibrolipomatus hamartoma of median nerve with coexisting macrodactyly (usually digit III)
Joint cavity and recesses	Effusions, synovial thickening, synovial hypervascularity, increased echogenicity of the synovial membrane, indicating chronic inflammation or fibrosis, and presence of loose bodies
Intra- and extra-articular fat tissue	Assessment of inflammation: increased echogenicity and increased vascularization
Hyaline cartilage of epiphyses, subchondral layer	Signs of damage (chondromalacia, erosions, cysts, and scars after injuries)
Growth plate area	Assessment in terms of post-traumatic lesions: features of exfoliation, erosions, and neoplasms, commonly found at the border of epiphysis and metaphysis
Ligaments	Assessment for post-traumatic changes
Bursae	Effusions, synovial thickening, hypervascularity, and loose bodies
Structures characteristic of a given joint or area (e.g., labrum in the shoulder or hip joint, menisci in the knee joint, etc.)	Limited diagnostic possibilities. The advantage of an MRI is unquestionable, but it is worth to at least make an initial diagnosis and recommend further examinations.

## Data Availability

Not applicable.
